# Mental health education for physiotherapists: A scoping review

**DOI:** 10.4102/sajpsychiatry.v29i0.2127

**Published:** 2023-12-01

**Authors:** Marilyn Hooblaul, Thayananthee Nadasan, Olagbegi M. Oladapo

**Affiliations:** 1 Faculty of Physiotherapy, Ekuhlengeni Psychiatric Hospital, KwaZulu-Natal Department of Health, Umbogintwini, South Africa; 2 Department of Physiotherapy, Faculty of Health Sciences, University of KwaZulu-Natal, Durban, South Africa

**Keywords:** mental health, physiotherapy, knowledge, attitude, perceptions

## Abstract

**Background:**

Physiotherapists play an integral role in the management of people living with a mental illness, yet little is known about their knowledge, attitudes and perceptions about mental health globally and particularly in South Africa.

**Aim:**

The purpose of the study is to map global evidence on mental health education for physiotherapists, including their knowledge, attitude and perceptions, with the goal of using this information to design an improved undergraduate curriculum for physiotherapy in South Africa.

**Setting:**

The search was focused on the South African and global context, with the participants as physiotherapists and physiotherapy students.

**Methods:**

The methodical framework proposed by Arksey and O’Malleys guided the scoping review. The online search used five electronic databases. An expert librarian assisted in the search strategy. English language, primary research articles that investigated physiotherapist or physiotherapy students’ knowledge, attitude and perceptions towards mental health were sought.

**Results:**

The search strategy extracted 226 published studies and 15 studies were included in the analysis. The results indicated that globally physiotherapists and physiotherapy students had limited knowledge about mental health. Improved attitudes were noted with a mental health training intervention. Negative perceptions were associated with limited knowledge.

**Conclusion:**

There was limited literature on the influence of education on mental health in physiotherapy on attitudes and perceptions. Physiotherapists desired more knowledge about mental health because of the prevalence of mental health disorders.

**Contribution:**

Because of the increasing prevalence of mental illness globally, the findings of this review suggest the necessity of integrating mental health content in the physiotherapists’ undergraduate programme to provide high-quality care physiotherapy management for people with mental illnesses.

## Introduction

One in six South Africans suffer from depression, anxiety or substance use disorders, and 40% of South Africans with human immunodeficiency virus (HIV) have a mental illness.^1^ The mental health crisis in South Africa is in dire need of change, with a lack of access and poor quality of mental health services.^2^ According to the Lancet Commission Report on Mental Health and Sustainable Development, the lack of mental health services is not unique to South Africa. Mental health services have been neglected globally.^3^ The quality of mental health services is worse than the quality of services for physical health.^3^ An integrated collaboration care model for people living with mental illness (PLWMI) and physical comorbidities can improve both mental and physical health outcomes with a patient-centred approach.^4^

Physiotherapy is a healthcare profession that deals with human movement and maximising functional physical potential. Physiotherapists use promotion, prevention, treatment interventions and rehabilitation strategies to promote, maintain and restore patients’ physical, psychological and social well-being.^5^ The key components of physiotherapy management in mental health include health promotion, preventive healthcare, treatment and rehabilitation for individuals or groups. Physiotherapeutic interventions include exercise, physical activity (PA), relaxation therapy, basic body awareness therapy (BBAT) and touch therapy.^6^ They create a therapeutic relationship in a supportive environment by applying the biopsychosocial model, with the goal of promoting functional movement, movement awareness, PA and exercises, bringing together the mental and physical aspects.^7^ The biopsychosocial model of health considers the biological (genetics, injury, disease, exercise, diet), social (family, school, relationship, poverty) and psychological (feelings, emotional intelligence, behaviour, beliefs) factors that affect the treatment outcomes.^8,9^ The scope of practice allows physiotherapists to manage patients with physical conditions and use the biopsychosocial approach in a person-centred manner. An integrated collaboration care model for PLWMI and physical comorbidities can improve both mental and physical health outcomes with a patient-centred approach.^4^ Bridging the collaborative gap between mental and physical health can be accomplished by improving access to care, reducing stigma and promoting policy of integrating physical and mental healthcare.^10^

Strong evidence exists to support the link between physical and mental health.^11,12,13,14^ Prince et al. (2000) further stated that the relationship between physical and mental health is very evident in patients with HIV, cardiovascular disease and diabetes. Physiotherapy in mental health is often regarded as insignificant in the management of PLWMI, and the role and value are unclear among the members of the multidisciplinary team (MDT).^15^ However, physiotherapy interventions complement medication and psychotherapy.^16^ The intervention of physiotherapists, which is associated with increased PA, can greatly enhance the quality of life for people with serious mental illness (SMI).^17^ In addition, physiotherapists (PT) can play an important role in addressing psychological factors when they are involved in the treatment and rehabilitation process of persons with disabilities, thereby enhancing rehabilitation outcomes.^18^ The role of the physiotherapist in the psychiatric MDT is to act as a bridge between physical and mental health and they are integral in providing treatment for the physical health for PLWMI.^19^

One major barrier to physiotherapists’ delivery of quality of care to PLWMI is stigma. Probst and Skjaerven^6^ defined stigma as a mark of disgrace. Stigma is primarily observed in mental health. The definition of stigma is quite complex, and it is linked to a trait, involves negative judgement, is social rather than personal, does not reside with a person or attribute but is created in interaction with others and is not static.^20^ Stigma increases the burden on mental health disorders and affects community members, families of PLWMI and medical professionals.^21^ Stigmatised attitude and lack of knowledge can have a negative influence on interactions with PLWMI.^22^ Tessem et al.^23^ stated that supervised student practice enhances communication and being sensitive to PLWMI, they adapted to challenging situations and found solutions through critical reflection on stigmatising attitudes and behaviours.

People living with mental illness have a higher prevalence of metabolic syndromes than the rest of the population in sub-Saharan Africa.^24^ Metabolic syndromes (MetS) consist of a cluster of conditions including abdominal obesity, diabetes, dyslipidaemia and hypertension. Patients with MetS have a greater risk for reduced PA performance.^25^ Bridging the collaborative gap between mental and physical health can be accomplished by improving access to care, reducing stigma and promoting a policy of integrating physical and mental healthcare.^10^ Consequently, incorporating modules on mental health during undergraduate physiotherapy training would be a good way to start.

There is a dearth of literature globally about the influence of education on mental health in physiotherapy on attitudes and perceptions. Providing mental health education for physiotherapy students in the undergraduate programme or postgraduate level would greatly enhance the skills of future physiotherapists.^26^ Negative stereotypes, attitudes and perceptions of mental illness can compromise access of PLWMI to physiotherapy services.^26^ Education and direct contact with PLWMI can assist to combat the problem of stigmatisation and other obstacles to their access to quality physical healthcare.^27,28,29^

This study aims to map the existing literature in South Africa and globally on the influence of mental health education on the knowledge, attitudes and perceptions of physiotherapists and physiotherapy students using a scoping review approach. There is no scoping review conducted to date that has explored the knowledge, attitudes and perceptions of physiotherapists or physiotherapy students regarding mental health. The study also aims to gain insight into future framework design for the inclusion of mental health in the undergraduate physiotherapy programme.

## Methods

A scoping review was conducted following the five steps outlined by Arksey and O’Malley in their methodological framework.^30^ In accordance with this framework, the five steps used were identifying the research question; identifying relevant studies; study selection; charting the data and collating, summarising and reporting results.

### Identification of the research question

The overall aim of the study underpinning the scoping review was: ‘What influence does the education on mental health in the physiotherapy undergraduate programme have on the knowledge, attitude and perceptions of physiotherapists and physiotherapy students?’ The research questions were identified as follows:

1. Is mental health content included in the physiotherapy undergraduate curriculum in South Africa and globally?
2. Does knowledge about mental health influence the attitudes and perceptions of physiotherapists and physiotherapy students?
3. What is the importance of mental health content in the physiotherapy undergraduate programme?
4. Is the knowledge gained through the physiotherapy undergraduate programme adequate for physiotherapists to manage PLWMI?

The population, concept and context (PCC) for the study are defined in Table 1.

**TABLE 1:** Population, concept and context framework for the main review question.

Variable	Variable defined
Population	Physiotherapists and physiotherapy students
Concept	Mental health training, undergraduate and postgraduate level
Context	South Africa and globally

### Ethical considerations

The researchers obtained ethical approval from the Humanities and Social Sciences Research Ethics Committee, HSSREC, of the University of KwaZulu-Natal ethical committee (ethical approval number: HSSREC/00004701/2022).

### Identifying relevant studies

The following electronic databases were used to conduct a search for literature: Google Scholar, PubMed, Cochrane Library, Web of Science and MEDLINE from 01 January 2000 to 18 October 2022. A search strategy was conducted in consultation with an expert librarian, using the following keywords: ‘physiotherapy’, ‘physical therapy’, ‘physiotherapist’, ‘physiotherapy student’, ‘mental health’, ‘mental illness’, ‘psychological distress’, ‘psychiatric illness’, ‘psychiatry’, ‘undergraduate programme’, ‘training’, ‘curriculum’, ‘knowledge’, ‘beliefs’, ‘perceptions’, ‘beliefs’, ‘views’, ‘behaviour’ and ‘attitude’. Boolean terms ‘AND’, ‘OR’ and ‘NOT’ were used to separate keywords. An expert librarian at the University of KwaZulu-Natal was consulted during the literature search. Mendeley Reference Manager v2.80.1 was used to manage all the citations.

### Eligibility criteria

The PCC framework was used to select the appropriate research articles. The inclusion and exclusion criteria were developed to identify key areas of interest. Table 2 outlines the inclusion and exclusion criteria.

**TABLE 2:** Inclusion and exclusion criteria.

Inclusion criteria	Exclusion criteria
Peer-reviewed articles where full text was obtained	Review articles
Articles published between 2000 and October 2022	Articles before 2000
Articles published in English	Articles that could not be translated into English
Articles that focused on physiotherapists or physiotherapy students’ knowledge, attitude and beliefs towards mental health	Studies where full text articles could not be obtained
All study designs were included.	-

### Study selection

Eligible articles were identified and uploaded to the citation manager, Mendeley v2.80.1, and all duplicate articles were removed. Two independent reviewers screened the titles and abstracts as well as the full text screening of retrieved articles that were relevant to the research objectives. A third reviewer screened any excluded citations and resolved any differences between the two reviewers to make a final decision. The library service at the University of KwaZulu-Natal was used to access full text articles that were not open-access publications.

### Charting data

Data from the selected studies were extracted and recorded in a standardised data abstraction tool designed. The following data were extracted to answer the study’s question:

- Participant characteristics: sample population and gender (Table 3).
- Study characteristics: author information and publication year, aim/objectives of the study, setting or location of the study (Table 4).
- Findings: tools used to quantify knowledge, attitudes and perceptions, the mental health content training (Table 4).
- Outcomes and conclusions: relevant to the study’s objectives.

**TABLE 3:** Participant characteristics for the 15 studies.

No.	Study	Participants	Participants characteristics	Country	Years of experience and/or level of training
1	Almirón et al. (2020)	Physiotherapists	Female: 75.94% Male: 24.06% (*n* = 187) Mean age: 32.84 years	Paraguay	8.4 ± 7.30 years of experience
2	Andrew et al. (2019)	Physiotherapists	**Focus group and interview** Female: 68% Male: 32% (*n* = 31) Mean age: 32.84 years **Survey** Female: 86% Male: 14% (*n* = 57) Mean age: 38 years 21–63 years	Western Australia	Focus group and interview: Mean 10 years of experience Survey: mean (14) 1–38 years of experience
3	Bhise et al. (2016)	Physiotherapy students	Female : 88.3% Male: 11.7% (*n* = 94) Mean age: 20.6 years	India	Final year physiotherapy students
		Medical students	Female : 44.6% Male: 55.4% (*n* = 94) Mean age: 21.9 years		Final year medical students
4	Connaughton and Gibson (2016a)	Physiotherapists	Female: 75% Male: 25% (*n* = 75) Mean age: 36 years 21–66 years	Western Australia	Mean (9.1) 4–35 years of experience
5	Connaughton and Gibson (2016b)	Physiotherapy students	Female: 71% Male: 29% (*n* = 162) Mean age: 20 years 17–37 years	Australia and New Zealand	First, second and final year students
		Physiotherapy Clinical Education Managers	59% response rate 10 universities responded		
6	Dandridge et al. (2014)	Physiotherapy students	Female: 80% Male: 20% (*n* = 173) Mean age: 22 years	United Kingdom	First, second and final year students
7	Driver et al. (2020)	Physiotherapists	Female: 67.5% Male: 32.5 (*n* = 208) Mean age: 36.2 years 21 to over 65 years	Australia	Mean (13.5) to over 40 years
8	Hemmings and Soundy (2020)	Physiotherapists	Female: 100% (*n* = 2) 26 and 41 years	Birmingham, United Kingdom	Unknown
		Patient	Female: 37.5% Male: 62.5% (*n* = 8) 19–56 years		
9	Hooblaul et al. (2020)	Physiotherapists	Female: 85.5% Male: 14.5% (*n* = 124) Mean age: 38 years	KwaZulu-Natal, South Africa	1 to > 10 years
10	Karyczak et al. (2020)	Doctor of physical therapy students	Female: 85.7% Male: 14.3% (*n* = 7) Mean age: 30.43 years	United States	Unknown
11	Lennon et al. (2020)	Physiotherapists	Female: 84% Male: 16% (*n* = 316) 20 to > 60 years	Ireland	0 to > 10 years of experience
12	Overmeer et al. (2009)	Physical therapists	Female: 86% Male: 14% (*n* = 42) Mean age: 45.8 years	Sweden	0 to > 21 years of experience
		Patients	**Patients of the course participants** Female: 74% Male: 26% (*n* =142) Mean age: 47 years 19–65 years **Patients of the control condition** Female: 86% Male: 14% (*n* = 228) Mean age: 44 years 18–65 years		
13	Probst and Peuskens (2010)	Physiotherapy students	Female: 70% Male: 30% (*n* = 219) Mean age: 18.8 years 17–24 years	Belgium	First and second year students
		Non-medical students	Female: 62% Male: 38% (*n* = 112) Mean age: 20.2 years 17–28 years		Unknown
14	Prasanna et al. (2021)	Physiotherapists	Female: 56% Male: 44% (*n* = 100) Mean age unknown	India	0 to > 5 years of experience
15	Yildirim et al. (2015)	Physiotherapy students	Female: 49.2% Male: 50.8% (*n* = 524) Mean age: 21.2 years 17–29 years	Turkey	First to fourth year students

Note: Please see the full reference list of the article, Hooblaul M, Nadasan T, Oladapo OM. Mental health education for physiotherapists: A scoping review. S Afr J Psychiat. 2023;29(0), a2127. https://doi.org/10.4102/sajpsychiatry.v29i0.2127, for more information.

**TABLE 4:** Study characteristics and findings of the 15 studies.

No.	Study	Aims	Methods	Assessment tools used	Key findings	Recommendations
1	Almirón et al. (2020)	To determine physiotherapists’ knowledge about the provision of services for people with mental disorders. To determine the role of physiotherapeutic interventions for both physical and mental conditions for people with mental illness.	A descriptive, observational, cross-sectional and prospective study	Online survey using closed and open-ended questions	About 83% PTs reported having no previous experience in mental health (MH). About 62% reported a lack of knowledge to manage physical conditions this patient group. About 36% reported having confidence in communicating and interacting with PLWMI. Nearly 40% stated that they did not see any PLWMI in practice and this 24% accounted the reason being inadequate knowledge of treatment for PLWMI.	The need of training of PTs about mental health in the undergraduate curriculum
2	Andrew et al. (2019)	To determine physiotherapists’ perspective of factors that influences the provision of services to manage the physical well-being of people with severe and persistent mental illness.	Mixed-methods research design	Online survey using closed-ended, single and multiple choice questions Focus group and interviews	Limited knowledge about the role of physiotherapy in the physical well-being of people with SPMI. Limited theoretical and practical knowledge to manage people with SPMI. Limited referrals for PT. PTs expressed concerns about safety and their perceived barriers to treating people with SPMI. About 23% had experience in working in mental health. About 18% felt confident managing people with SPMI. And 90% reported needing more education about management of people with SPMI and 48% needed information on communication skills.	PTs do not engage with this patient group because of inadequate knowledge and lack of confidence. There needs to be an increase in the undergraduate and postgraduate education in mental health for PTs.
3	Bhise et al. (2016)	To determine and compare the attitudes towards psychiatry of final year physiotherapy students and final year medical students that have completed the psychiatry curriculum.	Cross-sectional study	Attitude towards psychiatry tool (ATP-30)	ATP-30 scores were higher with physiotherapy students (105.8) than the medical students (91.9), concluding that physiotherapy students had a more positive attitude towards psychiatry than the medical students. Female students had a relatively higher ATP than males. PT students had 30 h of theory lectures in medical psychology in first year and 32 h teaching in second year and 15 h of clinical exposure in psychiatry and they have exams and compulsory attendance of lectures. A well-planned curriculum with mental health content can develop a positive attitude towards psychiatry.	The study showed a big difference in the attitudes towards psychiatry because of the training received by the students. Medical students should have the same psychiatry curriculum as the physiotherapy students.
4	Connaughton and Gibson (2016a)	To evaluate the self-reported attitudes of physiotherapists working in Western Australia towards psychiatry. To evaluate physiotherapists’ perceptions of their undergraduate education in psychiatry and mental health.	Cross-sectional, mixed-methods study	Online survey using ATP-30 tool and open-ended questions	Generally positive attitude (ATP-30 score of 107.8). Females had a more positive attitude than males. PT that saw PLWMI more frequently had a more positive attitude than those that did not. Significantly more positive attitude for PTs that self-report experiencing mental illness than those that did not. PTs reported that they acquired their knowledge about MH after graduation. PTs lacked communication skills to engage with PLWMI. PTs wished they had been educated about their role in mental, signs and symptoms, communication skills, medications and their side effects and referral pathways.	Undergraduate programme needs some revision and a need for advance postgraduate training in psychiatry and mental health.
5	Connaughton and Gibson (2016b)	To explore the self-reported attitudes of physiotherapy students from the University of Notre Dame Australia towards mental illness and psychiatry. To explore the self-reflections of students on education about mental health. To explore the mental health content in physiotherapy programmes in Australia and New Zealand.	Cross-sectional, mixed-methods study	ATP-30 tool and open-ended questions Course content questionnaire	All students showed a generally positive attitude towards psychiatry (ATP-30 score of 103.1). Students that had clinical experience exhibited a more positive ATP-30 score than those that did not have any clinical experience. Females had a more positive ATP-30 score than males. Students wanted more information on the side effects of psychiatric medications, signs and symptoms, pathophysiology, communication skills and the roles of the multidisciplinary team members. The course content questionnaire found that psychiatry is a critical area of curricula.	Integrating psychiatric content throughout the physiotherapy curriculum will reinforce the practice of the biopsychosocial approach.
6	Dandridge et al. (2014)	To explore physiotherapy students’ attitudes, educational and personal experiences towards people with mental illness.	Cross-sectional cohort study	Online survey using closed and open-ended questions	About 75.7% of students want more education about MH. About 30% of students had concerns about not knowing how to interact with PLWMI, their concerns were around lack of knowledge about communication. Students highlighted a lack of understanding about MH (23%). About 21% reported difficulties in providing treatment services for PLWMI. Students highlighted issues about patient characteristics such as patients being aggressive or violent, having mood swing and unpredictable behaviour (12%). Students that knew a PLWMI had few concerns than students that did not know a PLWMI.	A need for more training and education in mental health and more placements in MH.
7	Driver et al. (2020)	To determine the training of physiotherapists about psychosocial strategies. To explore that benefits and need for receiving training about psychosocial strategies and the method of delivery for training.	Cross-sectional, concurrent, mixed-methods design	Online survey using open-ended questions	PTs had limited psych training in the undergraduate programme. A few PTs received some education postgrad through courses. Others received informal training through on-the-job learning, in-service training and interacting with members of the multidisciplinary team. PTs perceived that the benefits of more training would be increased knowledge that would benefit patients and improve the management of psychosocial issues. PTs reported that they would like specific practical training on strategies and knowledge that were physiotherapy-specific regarding assessment and management of psychosocial issues. And the preferred method of delivery is didactic, interactive and taught by an expert in the field.	The findings of the study provide a framework that could be used to revise the undergraduate programme or postgraduate courses with the focus on psychosocial strategies.
8	Hemmings and Soundy (2020)	To explore the experiences of people with co-morbid physical and mental health conditions and physiotherapists about physiotherapy in mental health. To identify barriers and facilitators to care.	A qualitative study using interpretive phenomenological analysis	Semi-structure interviews (patients) and focus groups (physiotherapists)	MHCUs highlighted that a positive physiotherapy experience can increase motivation and compliance to the programmes. The study emphasised great importance on communication and building patient-physiotherapist rapport. PTs should improve on active listening and holistic care. A lack of mental health awareness for physiotherapists. Participants reported a lack of knowledge about mental health. The study identified the importance of improving the education at the undergraduate and postgraduate level to improve confidence of physiotherapist when treating PLWMI.	The study identified the need for integration of physiotherapists into the mental health services. Increase the understanding of mental health for people in management and policymakers.
9	Hooblaul et al. (2020)	To determine the knowledge, attitude and perceptions of physiotherapists in the public sector employed in KwaZulu-Natal, South Africa, towards mental health. To determine the preparedness of physiotherapists during the undergraduate training to manage people with mental illness.	A mixed-methods study	ATP-30 tool and focus groups	The study showed that PTs had a positive attitude towards mental health with an ATP-30 score of 103.70 Females and males had the same ATP-30 scores. PTs that know someone that had a mental illness frequently saw patients with a mental illness than those that did not. PTs expressed that after graduation they were not prepared to manage PLWMI because of their limited knowledge received at the undergraduate level of training. The knowledge that they have gained about mental health has been through self-learning. PTS gained knowledge about their role on mental health, prevalence of mental illness and stigma after graduation. PTs highlighted their need for more training on the effects of mental health on physical health, communication strategies and referral processes.	Need for the review of the undergraduate curriculum so that knowledge about mental illness, communication strategies, medications and side effects can be implemented theoretically and practically in the undergraduate programme.
10	Karyczak et al. (2020)	To examine doctor of physical therapy students’ attitudes and knowledge after a structured educational field experience working with people with severe mental illness.	A qualitative study using interpretive phenomenological analysis	Semi-structured interviews	Four main themes emerged from the study: attitudinal changes, improvement in skills, increase in competence and greater focus on person centredness. The study showed a positive attitude of the participants towards patients with SMI. This was attributed to their undergraduate training and exposure to managing patients with SMI. Participants reported an improvement in communication skills as well as empathy to this patient population. Participants reported their boost in confidence to manage patients with SMI and a feeling of empowerment and the ability to practice what they had learnt. They reported they had a better understanding of a person centredness approach.	The service learning programmes have shown to improve professional skills and reducing stigmatising attitudes and allow the students to better understand patients with SMI. Providing a structured service learning programme in the undergraduate curriculum is beneficial to the students and the patients.
11	Lennon et al. (2020)	To investigate the current practice and opinions of Irish physiotherapists about the management of patients with psychological distress.	A cross-sectional survey design	Online survey using closed and open-ended questions	PTs reported that they encountered patients experiencing psychological stress daily (40%) and 41% encountered them weekly. About 95% of PTs reported that the ability to identify psychological distresses was because of their clinical experience. Only 49% assessed their patients for psychological distresses. About 61% of the PTs were confident in identifying signs and symptoms of psychological distresses. About 13% of PTs reported that the entry level training was beneficial in identifying psychological distresses. The 33% of PTs that had additional training in mental were able to differentiate normal psychological responses and mental health disorders; their confidence was higher and they placed a greater importance in acknowledging a patient’s psychological distress, than this PTs that had not had any additional education on mental health. Common themes regarding the treatment approaches with patients with psychological distress were education of the patient, focusing on the psychological aspect of the patient, communicating with other healthcare professionals and re-evaluating treatment plans and goals. The common themes that were identified regarding the improvements required to better manage patients with psychological distress were support services of physiotherapy, improve the access for referrals, enhance the relationship with multidisciplinary team and further education for PTs.	The need for further education at the undergraduate and postgraduate levels as the study highlighted the significance of education on practicing PTs.
12	Overmeer et al. (2009)	To investigate the beliefs, attitudes, knowledge, skills and behaviour after attending a biopsychosocial course.	Randomised controlled trail with a pre- and post-test design	Questionnaire to assess attitudes and beliefs using the Pain Attitudes and Beliefs Scale for physical therapists (PABS-PT), the Health Care Providers Pain and Impairment Relationship scale (HC-PAIRS) and patient vignettes	PTs had significant changes in attitude, beliefs, knowledge and skills after the 8-week university course. The attitudes and beliefs depicted a more biopsychosocial approach. The study showed that PTs were less inclined to believe that pain justifies disability and limitation of activities. Their knowledge and skills on psychosocial risk factors greatly improved. Patients’ perceptions on PTs behaviour pre- and post course was the same.	Focused training opportunity for PTs could change attitudes and beliefs to a more biopsychosocial direction. The study showed that the course had an influence on knowledge and skills for PTs to identify and manage psychosocial risk factors.
13	Probst and Peuskens (2010)	To examine the attitudes of Flemish students towards psychiatry and mental health.	An experimental quantitative study	Questionnaire using the ATP-30 tool	ATP-30 scores for the entire group of students were moderately positive (104.5). Non-medical students scored higher than PT students. Female students scored significantly higher than males. Students with experience in psychiatry scored significantly higher than those without experience. Second year students scored significantly higher after completing the psychiatric course than the first year that did not receive any training. Second year students that passed the course showed higher ATP-30 scores than those students that failed the course.	The fundamental aim of the education through the undergraduate programme should be one that aims to promote a positive towards PLWMI. This can be done by ensuring adequate knowledge about the physiotherapy management of PLWMI. Skills about mental health are essential in the general practice of PT.
14	Prasanna et al. (2021)	To assess the attitudes of physiotherapists interacting with people with mental illness.	A non-experimental quantitative study	Questionnaire using the Mental Illness Clinicians Attitude Scale (MICA-4)	About 68% of PTs had less than 5 years of working experience. The study revealed that the attitudes were 15% positive, 73% neutral and 12% negative. The study showed that the neutral attitude was because of the lack of exposure to psychiatry.	Training PT students at an early stage can be effective to improve knowledge and confidence. Important to prevent stigma by promoting this area of specialisation.
15	Yildirim et al. (2015)	To investigate the beliefs of Turkish physiotherapy students towards mental disorders.	A quantitative study	Questionnaire using the Beliefs towards Mental Illness (BMI)	Students showed a moderately positive belief towards mental illness. Males and female scores were significantly the same. There were no significant differences in scores according to the year of studying. No difference in scores between students that had exposure of students that had a mental problem that required treatment or consultation with a professional and those that did not have any exposure to a mental health problem. Students that had a relationship with a PLWMI had a more positive belief towards mental illness.	PTs need to be familiar with mental health both clinically and for research purposes and therefore institutions should plan new curricula training that will include courses and/or lecture and practical learning that will be related to psychiatry and mental health.

Note: Please see the full reference list of the article, Hooblaul M, Nadasan T, Oladapo OM. Mental health education for physiotherapists: A scoping review. S Afr J Psychiat. 2023;29(0), a2127. https://doi.org/10.4102/sajpsychiatry.v29i0.2127, for more information.

SPMI, serious and persistent mental illness; PT, physiotherapist; PLWMI, people living with mental illness; MHCUs, mental healthcare users; SMI, serious mental illness.

### Collating, summarising and reporting the results

To answer the study question, all the relevant data were summarised. The results of the study selection were presented in a flowchart diagram and a narrative summary of the finding has been presented.^31^ The Mixed Methods Appraisal Tool (MMAT) version 2018 was used to appraise the quality of the selected studies^32^ (Appendix 1).

### Study characteristics

Initial search strategies as described resulted in 226 studies being identified from the database searches. Studies were screened and a total of 128 duplicated studies were removed. Subsequently, 98 studies were further screened and 78 articles did not meet the inclusion criteria and were therefore excluded from the review (Figure 1).

**FIGURE 1:**
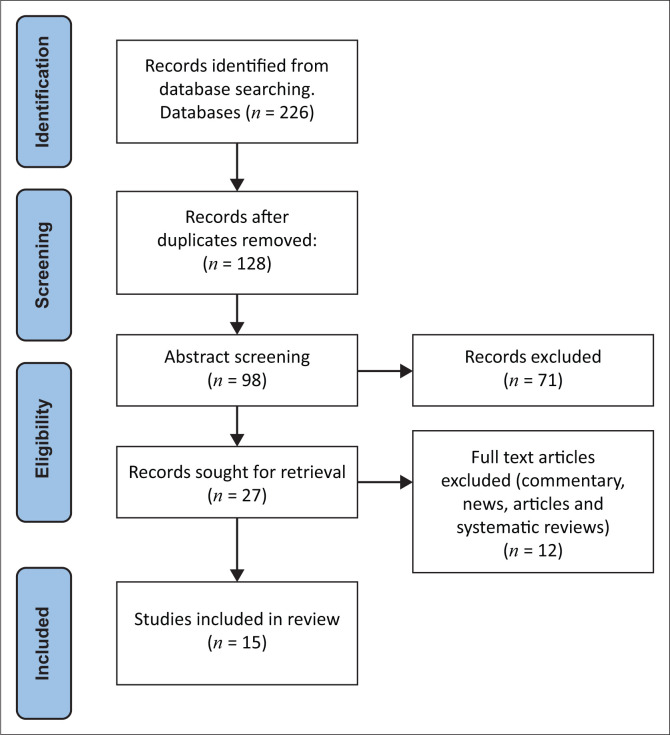
PRISMA flow diagram for the scoping review process.

The search produced 15 studies eligible for the scoping review. Table 3 summarises the study characteristics. Seven articles used a mixed-methods approach, four articles used a quantitative descriptive study approach, two articles used the qualitative study design and the remaining two studies used a quantitative randomised approach. Table 4 describes the study characteristics and the findings of the 15 studies; the study participants of the 9 studies were physiotherapists and the remainder (*n* = 6) were physiotherapy students. The majority of the participants in all 15 studies were female. Four studies were conducted within Australia. One study selected was conducted in the province of KwaZulu-Natal, South Africa. The themes of knowledge, attitude and behaviour regarding mental health were identified.

## Review findings

**FIGURE 2:**
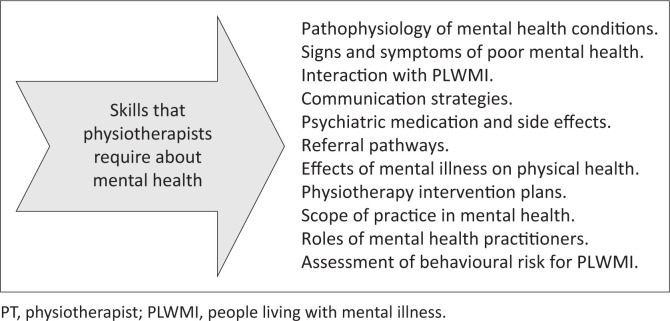
Knowledge about mental health.

Fifteen studies highlighted that there was limited training on mental health in the physiotherapy undergraduate programme.^26,33,34,35,36,37,38,39,40,41,42,43,44,45^ There was consensus among the studies that there is a need for more education about mental health. Participants specified areas of training about mental health that is of importance to them to effectively manage PLWMI (Figure 2). Areas of training included signs, symptoms and pathophysiology of mental health conditions, psychiatric medications and their side effects, physiotherapists’ scope of practice, physiotherapy interventions, the role of other members of the MDT and referral pathways. Skills training was conducted through didactic (lectures/, online modules/, reading) and interactive (practice/, case studies/, face-to-face) methods.

Four studies showed results on the effect of knowledge on attitudes, and participants exhibited positive attitudes towards psychiatry after completing training related to mental health.^34,40,42,43^ One study investigated the students’ knowledge using the ATP-30 tool to assess attitude after completing mental health training; even though the students’ attitudes were more positive than the medical students, it is unclear whether the training influenced a positive attitude.^43^ Probst and Peuskens^43^ study focused on the effect of a 67 h course on psychiatric rehabilitation on the students’ attitudes towards psychiatry. The study showed that the second year students had a significantly more positive attitude after the psychiatric rehabilitation course. The ATP-30 tool was used pre- and post-test, and therefore the knowledge gained through the training was shown to influence attitude positively. Karyczak et al.^40^ conducted a semi-structured interview with students after attending an 8-week service learning with individuals with SMI. The study showed that because of the knowledge gained, participants’ competence and skills were improved, and they had more confidence in managing individuals with SMI. Overmeer et al.^42^ focused on the psychosocial factors that affect pain by using a pre- and post-test design. People with chronic pain have a risk of developing mental health problems; it affects their sleep and increases stress and can lead to depression.^46^ The study found significant changes in the knowledge, attitude, beliefs and skills of physiotherapists after the course, and this was the only study that involved postgraduate training for physiotherapists. One study showed that a lack of exposure to psychiatry was the reason for the physiotherapists’ neutral attitude towards mental health and psychiatry.^44^ The participants were majority female, and 4 of the 15 studies showed that females had a more positive attitude than their male counterparts.

**FIGURE 3:**
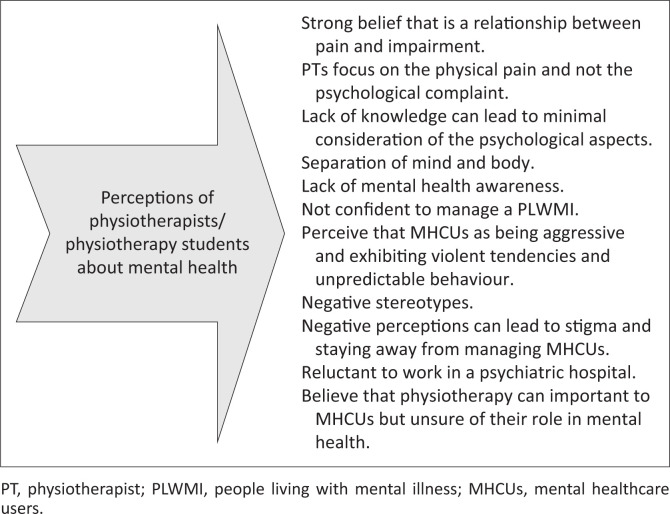
Perceptions about mental health.

Some common themes of perceptions are depicted in Figure 3. Some perceptions noted by participants were their neglect of psychological complaints, negative stereotypes, stigmatising behaviour, fear of safety and reluctance to work in a psychiatric hospital. Perceptions can be influenced by exposure to PLWMI. Physiotherapists that had limited knowledge perceived mental healthcare users (MHCUs) as a potential threat and tended to rely on their own personal beliefs. Table 5 describes the barriers and facilitators to managing PLWMI. The benefits of training on psychosocial strategies are increased knowledge, improvement in the assessment and management of psychosocial issues and the patients’ benefit.

**TABLE 5:** Barriers and facilitators to managing people living with mental illness.

Barriers	Facilitators
Lack of knowledge about mental health	Adequate knowledge about mental health
Inadequate communication skills	Implementation of the biopsychosocial model
Stigma	Ability to refer patients to the correct member of the MDT when required
Personal barriers such as negative attitudes and perceptions	Knowing someone with a mental illness
Lack of confidence	Consultation with a psychiatrist or psychologist
Fear of MHCUs	Experience with managing PLWMI
Perception of violent tendencies of MHCUs	Time to form a rapport with client
Lack of resources	Mental health awareness
Insufficient time with MHCU	Health promotion
Not being able to identify psychological distresses	In-service training and/or postgraduate training
Unsure of the roles of the multidisciplinary team and the correct referral pathways	-
Not understanding the scope of physiotherapy in mental health and treatment modalities	-
Separation of the body and mind	-

MDT, multidisciplinary team; MHCUs, mental healthcare users; PLWMI, people living with mental illness.

## Discussion

Knowledge can foster positive attitudes as proven in four studies with an intervention that involved mental health training.^34,40,42,43^ The four studies showed a considerable improvement in the attitudes of the physiotherapy students after the implementation of mental health training; therefore, the influence of education was positive on the attitudes of the students. Positive attitudes can foster empathy, reduce stigma, improve communication and enable physiotherapists to provide an integrated approach of care for PLWMI.

The reviewed studies did show physiotherapists and physiotherapy students having a positive attitude even though they self-reported having limited knowledge about mental health and psychiatry.^26,33,35,36,37,38,39,41,44,45^ Education on mental health can complement the positive attitudes by increasing their confidence, understanding, collaboration and capacity to provide holistic care. This will benefit the physiotherapists and PLWMI. Only a study conducted in South Africa noted that physiotherapists reported limited knowledge about mental health and highlighted the need for more education either in the undergraduate or postgraduate programme as they are encountering PLWMI with physical conditions.^39^ Participants gained their knowledge about mental health from colleagues of the MDT and from years of working experience. Belgium, Norway, Sweden and many European countries offer a programme in the area of physiotherapy in mental health, but it is unclear whether this is being done in any of the other provinces in South Africa except KwaZulu-Natal.^36^ The role of physiotherapy in mental health is evident, but there needs to be a strengthening of skills and knowledge to meet the needs of PLWMI.^33^ Education about mental health can act as an enabler to improving access to physiotherapy services, but limited knowledge and skills are seen as a barrier as physiotherapists tend to avoid the management of patients with conditions that are unfamiliar to them.^26^ It is evident that there is a disparity in the training being received at various universities globally. The review did not uncover a clear description of the mental health content in the physiotherapy undergraduate curriculum in South Africa and globally.

Adequate knowledge and positive attitudes can greatly reduce stigma. Negative perceptions and stereotypes lead to stigmatising behaviour. A perception that all MHCUs are volatile and dangerous can greatly affect access to care. Studies have shown that participants had a positive attitude towards psychiatry and mental health, yet they had negative perceptions and limited knowledge.^33,39^ Initiatives that can improve negative perceptions include engaging in direct interactions with MHCUs and providing education about mental health.^33^ Attitude is affected by knowledge gained and therefore attitude can influence behaviour.^47^ Because of the prevalence of mental illness and the limited knowledge of physiotherapists about mental health, physiotherapists had resorted to other avenues to bridge the knowledge gap so they may deliver an efficient and effective service to PLWMI. The inclusion of mental health training in the undergraduate training and postgraduate courses will reinforce the biopsychosocial model of approach to all patients. Training will benefit physiotherapists in managing patients holistically with mental illness and physical conditions. Attitudes are also influenced positively by knowing an individual with mental illness, having consulted a mental health professional or the increased frequency of managing PLWMI.^35,36,39^ More clinical experience influenced attitudes more positively. Clinical experience with patient interaction can deter negative stereotypes and perceptions.^26^

Basic knowledge about mental health and mental illnesses would greatly improve attitudes. Negative attitudes after graduation may not do justice to PLWMI in general, as psychological factors play an important role in rehabilitation of physical disorders.^31^ A well-planned course on mental health for physiotherapy students shows significant improvements in beliefs and attitudes but more so on knowledge and skill providing a mutual benefit for the student and the patient.^34,40^ Because of the growing prevalence of mental illness and comorbidities globally, physiotherapists are managing PLWMI. Consequently, it is essential that they receive education about mental health conditions and the role of physiotherapy in mental health in the undergraduate programme.^35,36,39,48^ The perception that all MHCUs are violently aggressive is perpetuated by social media.^39,49^ Mental health awareness education can reduce stigma and kerb the fear of interacting with MHCUs, which is one of the ways to facilitate the management of PLWMI^38^ as seen in Table 5.

## Limitations

The researcher focused on the influence of education on mental health on knowledge, attitudes and perceptions of physiotherapist and physiotherapy students; well-known electronic databases were used to conduct the search. Although the scoping review applied a rigorous and systematic search strategy, that identified several studies; it is acknowledged that some relevant studies might have been omitted as they were published in other languages besides English. Many of the studies used different outcome measures and study designs and this made it difficult to compare studies. There were inconsistencies in the outcome measures of the studies, and these show a discrepancy in the use of following gold standards for physiotherapy.

## Conclusion

To our knowledge this is the first review that explored published research about the influence of mental health training in the physiotherapy undergraduate programme has on knowledge, attitude and perceptions of physiotherapists and physiotherapy students. There was one study conducted in South Africa, which found similar results as the international studies showing limited knowledge and underpreparedness of physiotherapists to manage PLWMI. Knowledge was acquired through learning experiences, in-service training and interactions with members of the MDT. These studies also found common findings about attitude and perceptions. Limited knowledge about mental health did not affect attitude, as physiotherapists and physiotherapy students showed a positive attitude. Knowledge, however, had an influence on perceptions, beliefs and views about mental health. Perceptions were negative and depicted by personal experiences and social media. This leads to increased stigma and the forming of barriers to care. Physiotherapy students require a good foundation about their role in mental health and emphasising the biopsychosocial model. Because interventions of mental health training were associated with improved attitudes, it is therefore logical to assume that optimal foundation regarding mental health can engender a more positive attitude in physiotherapists and highlighting the importance of mental health content in the undergraduate programme. Many of the studies showed the limited mental health content. There is an increase in prevalence of mental health disorders globally, and there is a need for the integration of mental health content in the undergraduate curriculum. The scoping review recommends the review of physiotherapy curriculum to include mental health content.

## Figures and Tables

**FIGURE 1-A1:**
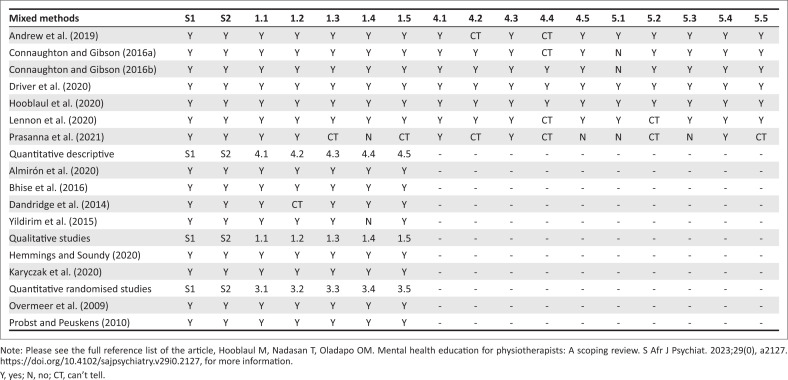
Mixed Methods Appraisal Tool critical appraisal checklist.
